# Repolarization time map in catheter ablation for scar‐related reentrant ventricular tachycardia

**DOI:** 10.1002/joa3.70070

**Published:** 2025-04-15

**Authors:** Naoya Kataoka, Teruhiko Imamura, Takahisa Koi, Keisuke Uchida, Koichiro Kinugawa

**Affiliations:** ^1^ Second Department of Internal Medicine University of Toyama Toyama Toyama Japan

**Keywords:** conduction delay, repolarization time, scar‐related ventricular tachycardia

## Abstract

**Background:**

Ventricular tachycardias (VTs) associated with scar tissue involve reentry mechanisms influenced by both conduction abnormalities and repolarization heterogeneity. However, existing mapping techniques have predominantly focused on conduction delay.

**Methods:**

This retrospective study analyzed 33 consecutive cases of catheter ablation for sustained VT. The EnSite system was employed to measure repolarization time (RT) with a high‐pass filter setting of 0.05 Hz. We compared the characteristics and concordance rates of short RT areas, defined as white or red‐colored regions, with those identified through conventional mappings in relation to ablation targets. These short RT areas were defined based on the longest interval from the QRS onset to the maximal *dV*/*dt* point of unipolar potentials, which was divided into eight equal segments.

**Results:**

Out of 31 VTs across 26 cases, we found that 18 (58%) of the identified ablation targets corresponded to deceleration zones (DZs). Of them, 16 (89%) also overlapped with areas of short RTs. Notably, among the remaining 13 VTs without ablation targets corresponding to DZs, 9 (69%) had ablation targets located in areas with short RTs. The distribution analysis revealed that 84% of short RT regions were located near the exit site, whereas 75% of DZs were situated near the entrance site. The distance between the two was 16 mm (interquartile range: 6.5–27.5 mm).

**Conclusion:**

This study underscored the potential of RT mapping in identifying ablation targets in scar‐related VTs. Incorporating both repolarization heterogeneity and conduction delay could significantly enhance the understanding of the intricate circuits involved in these arrhythmias.

## Graphical abstract

Repolarization heterogeneity plays a crucial role in scar‐related reentrant ventricular tachycardia. Repolarization time (RT) mapping using unipolar potential recordings with a high‐pass filter setting of 0.05 Hz enables visualization of these heterogeneities, and regions with shortened RT may serve as potential targets for ablation.

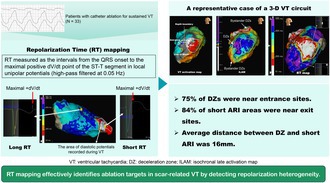

## 1 INTRODUCTION

Catheter ablation for ventricular tachycardias (VTs) associated with scar tissue and the surrounding border zone remains challenging, despite advancements in multiple electrophysiological three‐dimensional mapping techniques in the contemporary era.^1^, ^2^ One major contributing factor to these challenges is the acknowledged hemodynamic instability during VTs, which complicates the construction of activation mapping for identifying critical isthmuses.^3^ One approach to addressing the hemodynamic issue is the use of mechanical circulatory support during VT activation mapping. However, this method has not demonstrated superiority compared to procedures conducted without the utilization of mechanical circulatory support in current literature.^4^ Further new techniques for detecting critical isthmuses during sinus rhythm are warranted.

In the scar tissue of myocardial infarction, residual myocytes demonstrate heterogeneity in action potential durations, which leads to reentrant tachycardias.^5^ The durations of action potentials in cardiomyocytes cannot be directly measured in vivo in humans; however, repolarization time (RT) or activation recovery interval, obtained from electrocardiograms, may serve as indicators of action potential durations.^6^ We recently reported a case of ischemic cardiomyopathy, demonstrating that assessing activation recovery interval via local unipolar potentials utilizing the Wyatt method visualized the heterogeneity of action potential durations and identified the critical isthmus within the VT circuit.^7^

This cohort study aimed to investigate: (1) whether RT mapping could effectively identify the ablation targets of VT circuits across multiple cases, and (2) the differences in the location of short RT areas within VT circuits compared to the area of interest identified by conventional substrate mapping.

## 2 METHODS

### 2.1 Study population

In this retrospective cohort study, consecutive cases of catheter ablation for sustained VTs at our institution between January 2019 and August 2024 were screened. Subjects who met the following criteria were finally enrolled: (1) underwent high‐density electro‐anatomical maps using Advisor HD Grid Mapping Catheter Sensor Enabled (Abbott, MN) and (2) were diagnosed with scar‐related reentrant VTs.^8^ Eligibility for enrollment in this study was not affected by whether the diseased chamber is the right or the left ventricle. Procedures conducted without high‐density mapping during the baseline rhythm were excluded. Cases with atrial fibrillation were also excluded as the irregular R–R intervals complicate the measurement of refractory periods. The present study was approved by the institutional review board at the University of Toyama. Informed consent was waived given the retrospective nature without any intended interventions and opt‐out method.

### 2.2 VT induction

All subjects underwent catheter ablation utilizing the EnSite system (Abbott, MN) under deep sedation with propofol and dexmedetomidine. The transseptal puncture was adopted for the left ventricular approach, and heparin was infused intravenously with the target‐activated clotting time >300 s. Following the induction of VTs, either with or without isoproterenol infusion, ventricular pacing from the right ventricle was performed for the resetting and entrainment of VTs to facilitate the diagnosis of reentrant tachycardias.^9^ Subsequently, activation maps utilizing the Advisor HD Grid were performed for hemodynamically mappable VTs. In cases where the VTs were hemodynamically unstable, termination was achieved through rapid pacing or direct current cardioversion. VTs were induced using programmed stimulation from the right or the left ventricle at two base cycle lengths (400 and 600 ms), with up to three extrastimuli decremented to ventricular refractoriness. Subsequently, three‐dimensional electroanatomical mapping utilizing the Advisor HD Grid was performed during sinus rhythm or biventricular pacing rhythm.

### 2.3 The method for determining ablation targets

The ablation targets were identified based on the following criteria: (1) For cases with stable hemodynamics, sites exhibiting diastolic potentials during VT and where programmed stimulation confirmed the presence of the VT isthmus; and (2) For cases with unstable hemodynamics, sites where pace mapping reproduced the originally induced VT waveform, delayed potentials were recorded during sinus rhythm or ventricular pacing, and ablation rendered VT non‐inducible. All enrolled cases were assessed for the isochronal crowding zones using the isochronal late activation map (ILAM).

### 2.4 Functional substrate mapping

The following four distinct pieces of information were obtained. (1) Peak‐toto‐peak amplitudes were measured to evaluate low‐voltage areas, defined as regions with unipolar voltages less than 8.3 mV in the left ventricle and 5.5 mV in the right ventricle; (2) as areas with bipolar voltages less than 1.5 mV, as previously reported^10^, ^11^; (3) ILAM was created using bipolar potentials, with annotations for the last deflection, facilitating the identification of the deceleration zones (DZs), which are defined as areas containing more than three isochrones within a 1 cm radius^1^; and (4) the RT mapping consisted of the unipolar morphologies.

The RT mapping was conducted using unipolar morphologies with an indifferent electrode positioned in the inferior vena cava.^7^ The local RT was defined as the interval from the surface QRS onset to the point of maximal *dV*/*dt* in the J‐ST phase of the unipolar potentials. The procedure for configuring the EnSite system was as follows:

1. The high‐pass filter for the catheter unipolar signals in the waveforms was set to “0.05 Hz”.
2. The map polarity was adjusted to “Unipole”.
3. In the MAP settings, the sweep speed was configured to 100 mm, and the independent scoring interval was adjusted from the QRS onset to the end of the T wave.
4. The reference [MultiECG] was aligned with the offset of the surface QRS, and the end of the window was set to 100 msec after the T wave's termination. The start of the window was gradually shifted backward for each case to ensure proper coverage of the local fragmented potentials in the unipolar leads (i.e., delayed local depolarizations). Although our initial case report measured the activation recovery interval in a case of scar‐related reentrant VT, the starting point varies across cases, resulting in a measurement method that deviates from the original definition of activation recovery interval.^7^ Therefore, in this study, we chose to use the QRS onset as the starting point for measuring RT.
5. The detection was set to “+*dV*/*dt*”, consistent with the Wyatt method.^12^
6. The SCORE was configured to the high 80s.
7. The Auto Outlier Filtering function was employed to automatically remove outlier points.
8. Finally, the longest interval from the QRS onset to the maximal *dV*/*dt* in the J‐ST segment was divided into eight equal segments, each represented by a distinct color.

Warm colors, beginning with white, were associated with short RT, whereas cool colors corresponded to long RT. The short RT area was defined as white‐ or red‐colored regions on the RT map. Since short RT areas were correlated with the VT isthmus in our case report, they were designated as areas of interest.^7^

### 2.5 Study endpoints

To assess the characteristics of the RT map, the following three points were evaluated: (1) the comparison of the sizes of areas of interest identified by each substrate mapping technique, (2) the concordance rates between the areas of interest and the ablation targets for each method, and (3) the differences in distribution within the ablation targets between the DZs and the short RT areas, specifically concerning the locations of the short RT areas at the entrance, mid‐portion, or exit site of the VT circuit.

Subsequently, the recurrence of VTs was assessed during a 1‐year follow‐up. The clinical significance of radiofrequency ablation coinciding with areas of short RTs was evaluated and compared to cases where ablation coincided with DZs identified by ILAM.

### 2.6 Statistical analysis

Continuous data were described as the median with the interquartile range. A Friedman's test was conducted to compare the low‐voltage area, DZ, and short RT area, as these comparisons were paired. The Steel‐Dwass test was subsequently employed as a post‐hoc analysis. The Kaplan–Meier curves were generated to evaluate the incidence of VT recurrence, and the Log‐rank test was employed to compare cases with and without ablation of the areas of interest. A two‐sided *p*‐value <.05 was considered statistically significant. Data analyses were performed using JMP ver. 14 (SAS, NC, USA).

## 3 RESULTS

### 3.1 Subject characteristics

Among the 33 cases initially screened, 7 were excluded due to the presence of polymorphic, focal VTs, or the use of alternative three‐dimensional mapping systems. The remaining 26 cases, comprising a total of 43 induced VTs, were subsequently evaluated. After excluding insufficiently assessed for reentry, 31 VTs (median cycle length [interquartile range]: 340 [290–380] ms) were ultimately included in the analysis. Patient characteristics are summarized in Table 1. The median age of the cohort was 75 years, with 20 patients (77%) being male. Ischemic etiology was the most common, observed in nine patients (35%), followed by dilated cardiomyopathy in six patients (23%), arrhythmogenic ventricular cardiomyopathy in three patients (12%), and congenital heart disease in three patients (12%). The median PAINESD risk score was eight points, with 15 patients (58%) classified as low risk, 7 patients (27%) as intermediate risk, and 4 patients (15%) as high risk.^13^

**TABLE 1:** Patient characteristics.

Variable	
Age, years	75 [57–82]
Male, *n* (%)	20 (77)
Body mass index, kg/m^2^	23.7 [20.0–26.1]
Left ventricular ejection fraction (%)	37 [30–54]
Underlying cardiac disease	
Ischemia, *n* (%)	9 (35)
Dilated cardiomyopathy, *n* (%)	6 (23)
Arrhythmogenic ventricular cardiomyopathy, *n* (%)	3 (12)
Congenital heart disease, *n* (%)	3 (12)
Sarcoidosis, *n* (%)	2 (8)
Amyloidosis, *n* (%)	2 (8)
Hypertrophic cardiomyopathy, *n* (%)	1 (4)
Cardiac implantable electronic devices, *n* (%)	18 (73)
Implantable cardioverter defibrillator, *n* (%)	14 (54)
Cardiac resynchronization therapy with a defibrillator, *n* (%)	6 (23)
Medications	
ACEi, ARB, or ARNI, *n* (%)	18 (69)
β‐Blockers, *n* (%)	19 (73)
Mineralocorticoid receptor antagonists, *n* (%)	14 (54)
Sodium‐glucose cotransporter 2 inhibitor, *n* (%)	12 (46)
Loop diuretics, *n* (%)	16 (62)
Inotropes, *n* (%)	5 (19)
Amiodarone, *n* (%)	21 (81)
Lidocaine, *n* (%)	7 (27)
PAINESD risk score, point	8 [4–13]
Low risk, *n* (%)	15 (58)
Intermediate risk, *n* (%)	7 (27)
High risk, *n* (%)	4 (15)

Abbreviations: ACEi, angiotensin‐converting enzyme inhibitor; ARB, angiotensin receptor blocker; ARNI, angiotensin receptor neprilysin inhibitor.

Details of the procedures are outlined in Table 2. Of the 26 total procedures, six were redo sessions. Substrate mapping was performed in 21 cases under sinus rhythm, while the remaining five cases were conducted under biventricular pacing rhythm. Four VTs in three cases of arrhythmogenic ventricular cardiomyopathy originated from the right ventricle. The median procedural time was 3 h, with or without epicardial mapping (performed in six cases). Complications requiring treatment occurred in three cases, all of which involved bleeding necessitating transfusion.

**TABLE 2:** Details of procedures.

Variable	Overall
First session, *n* (%)	20 (77)
Redo session, *n* (%)	6 (23)
Cardiac rhythm during substrate mapping	
Sinus rhythm, *n* (%)	21 (81)
Bi‐ventricular pacing rhythm, *n* (%)	5 (19)
Origins of ventricular tachycardia	
The right ventricle, *n* (%)	4 (13)
The left ventricle, *n* (%)	27 (87)
Total procedural time, hours	3 [2.5–4.0]
Requiring the epicardial mapping, *n* (%)	6 (23)
Complications requiring treatment, *n* (%)	3 (12)

### 3.2 Comparison of the areas of interest identified by each method

In functional substrate mapping, both bipolar and unipolar low‐voltage areas were identified in all cases; however, the DZ in ILAM was identified in 21 cases (81%). The RT map provided a relative assessment of local action potential durations in each case, enabling the identification of regions with short RT, which were displayed in white or red across all cases.

**FIGURE 1:**
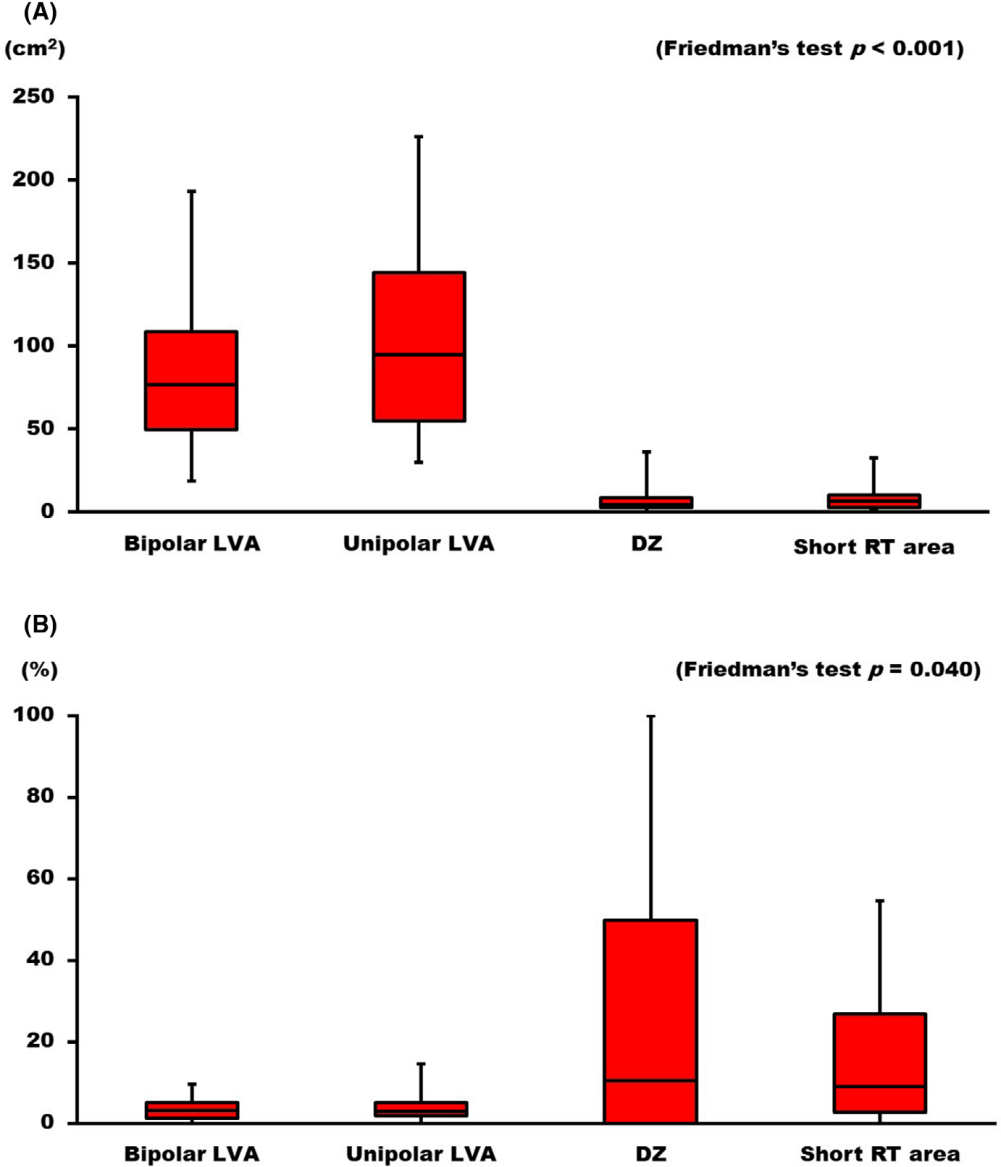
Box plots of the areas of interest and the concordance rates with the ventricular tachycardia (VT) isthmus. (A) The areas of interest for each method: From left to right, the bars represent the bipolar low‐voltage area (LVA), unipolar LVA, deceleration zones (DZ) identified by isochronal late activation mapping (ILAM), and regions with short repolarization times (RT). (B) The concordance rates between the region of interest and the VT isthmus: The arrangement of the figures and abbreviations is the same as in (A).

The sizes of the areas of interest in each method, as well as the concordance rates between the ablation targets and the areas of interest in each method, were compared. On a per‐case basis, the areas with short RT, with a median size of 5.1 (2.9–10.1) cm^2^, were comparable to the DZ areas at 4.2 (2.8–8.4) cm^2^; however, they were significantly smaller than the bipolar low‐voltage areas, which had a median size of 76.7 (49.2–108.4) cm^2^, and the unipolar low‐voltage areas, which measured 94.4 (54.6–144.0) cm^2^ (*p* < .001, Figure 1A). The concordance rates for both the bipolar and unipolar low‐voltage areas were significantly lower than those for the DZ and short RT areas (DZ vs. Uni LVA, DZ vs. Bi LVA, *p* = .005 for both; and Short RT vs. Bi LVA and Short RT vs. Uni LVA, *p* < .001 for both, Figure 1B).

### 3.3 Representative cases

**FIGURE 2:**
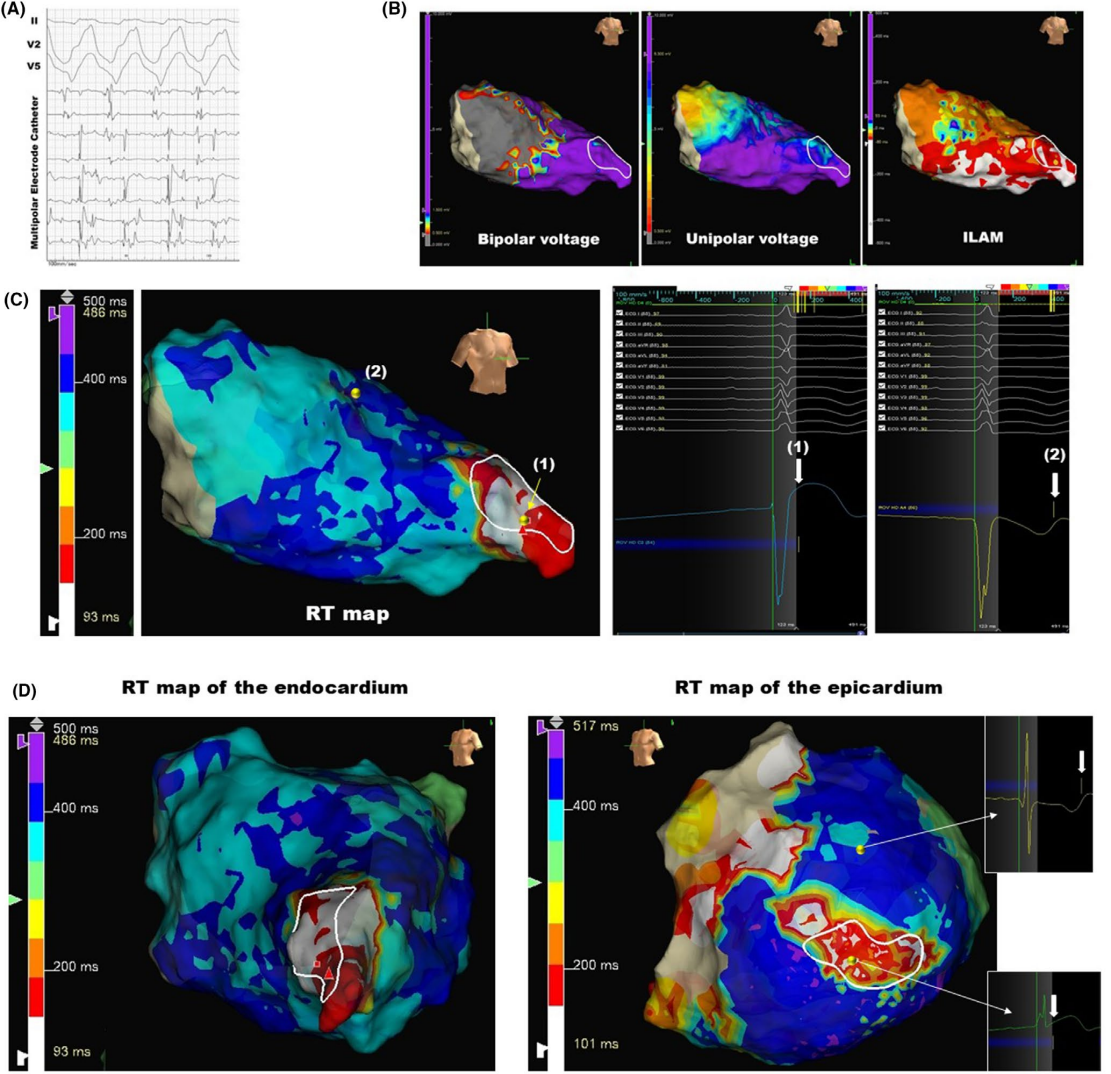
A representative case of complete concordance between the short RT region and the VT isthmus. (A) Intracardiac electrocardiogram during VT in a patient with hypertrophic cardiomyopathy. A multipolar electrode catheter (the Adviser HD Grid) positioned at the left ventricular apex, shown as a white circle in panel (B). (B) The geometries display the RAO view of the left ventricle. (C) RT map; the unipolar morphologies at the point of the shortest RT (1) and the point of the latest RT (2). White arrows indicate the maximal *dV*/*dt* point of the J‐ST segment. (D) Comparison of the endocardial and epicardial RT maps. The left panel illustrates the endocardial map, while the right panel depicts the epicardial map, both corresponding to the Left Anterior Oblique 45° view. RAO, right anterior oblique; RT, repolarization times; VT, ventricular tachycardia.

A representative case in which the ablation target corresponded to the short RT area without any DZs is shown in Figure 2. A 72‐year‐old female diagnosed with hypertrophic cardiomyopathy underwent catheter ablation for sustained VT. Clinical VT was induced by triple extrastimulation in the left ventricle, and diastolic potentials were recorded during the tachycardia using a multipolar electrode catheter (Figure 2A). These diastolic potentials were identified at the left ventricular apex, as indicated by the white circle in Figure 2B. Substrate mapping during sinus rhythm demonstrated a scar area in the basal septum and a scar border zone in the mid‐septum extending to the anterior wall. Conduction delay was identified as a DZ in the septum within the ILAM; however, this did not correspond with the ablation target. Notably, the RT map successfully identified the ablation target as a short RT area, indicated by white or red coloration (Figure 2C). The unipolar morphology in the long RT area exhibited a flat or down‐sloping ST segment with a negative T wave (Figure 2C, (2)); however, the morphology in the short RT area demonstrated an up‐sloping ST segment without a negative T wave (Figure 2C, (1)). The epicardial RT mapping was also obtained in this case. The contralateral region to the endocardial short RT area also exhibited relatively shorter RT values compared to those in other regions of the epicardium (Figure 2D).

**FIGURE 3:**
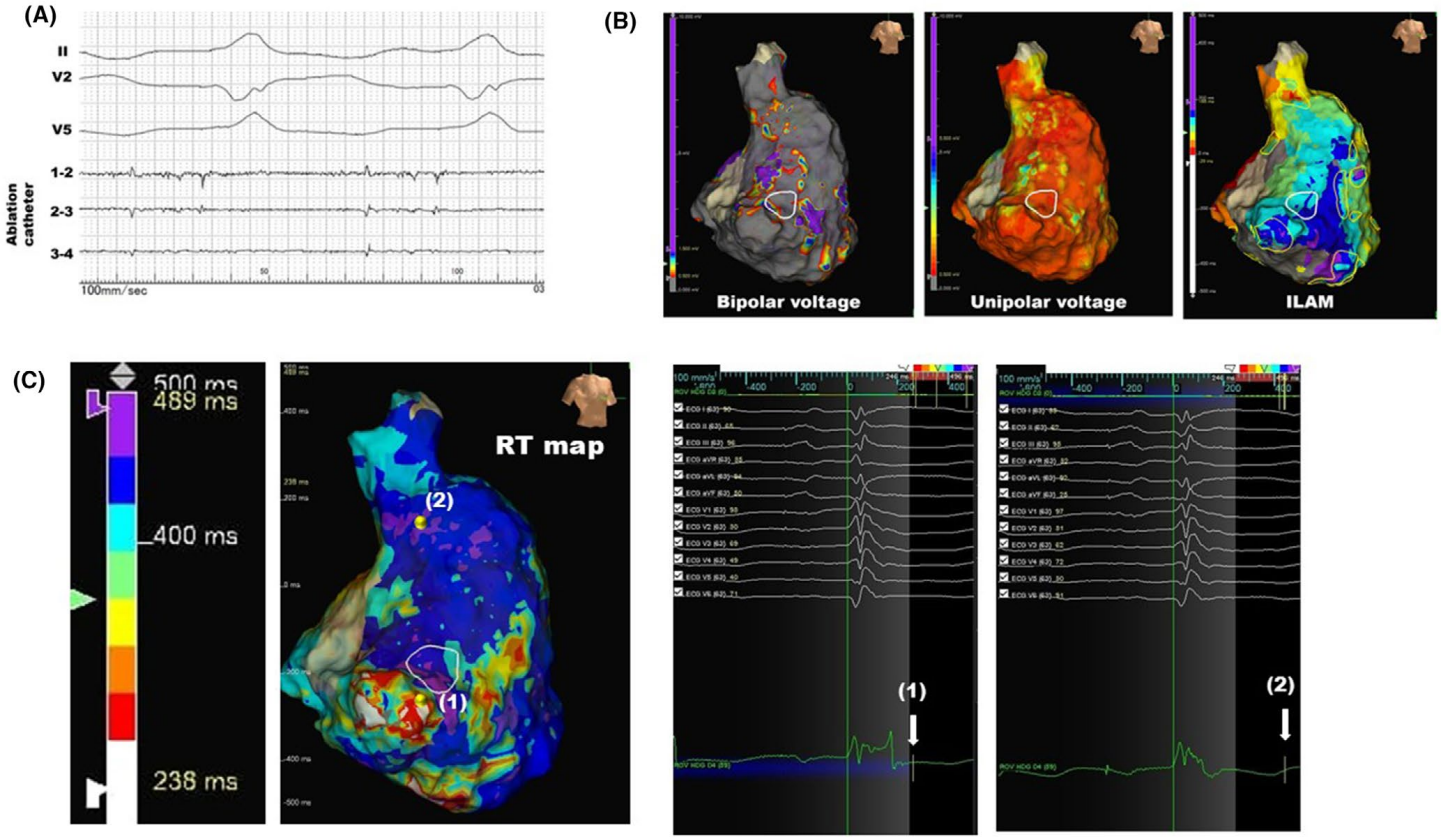
A representative case of adjacent short RT area and DZ. (A) Intracardiac electrocardiogram during VT in a patient with arrhythmogenic ventricular cardiomyopathy. The ablation catheter is positioned at the lateral site of the tricuspid annulus, shown as a white circle in panel (B). (B) The geometries display the RAO view of the right ventricle. (C) RT map; the unipolar morphologies at the point of the shortest RT (1) and the point of the latest RT (2). White arrows indicate the termination of the RT. DZ, deceleration zone; RAO, right anterior oblique; RT, repolarization times; VT, ventricular tachycardia.

The second case demonstrated a generally lower degree of variability in RT compared to the first case (Figure 3). This case is a 53‐year‐old male diagnosed with arrhythmogenic ventricular cardiomyopathy. The ablation catheter recorded mid‐diastolic potentials during VT at the location indicated by the white circle in Figure 3B, as illustrated in Figure 3A. Broad bipolar and unipolar low‐voltage areas were identified at the lateral aspect of the right ventricle (Figure 3B). The DZs identified by ILAM represented as yellow circles were located adjacent to the ablation target (white circle). The region with short RT, depicted in red or white, was also adjacent to the ablation target and corresponded with the DZs. As shown by the color bar representing RT, this case exhibited a longer RT in the shortest region, measuring 238 ms compared to 93 ms in the first case. However, the longest RT in both cases was nearly identical, at approximately 490 ms, suggesting a smaller degree of variability in RT in the second case.

### 3.4 Position of short RT area within VT circuit

The utility of detecting ablation targets was evaluated across all 31 VTs. A total of 18 (58%) DZs corresponded to ablation targets. Among these, 16 (89%) also overlapped with areas of short RTs. For the remaining 13 VTs, ablation targets in 9 (69%) corresponded to short RT areas. Notably, 58% of the ablation targets corresponded to DZs, whereas 81% corresponded to short RT areas. The correspondence rate was similar between the endocardium and the epicardium [17 (85%) vs. 4 (67%), *p* = .318]. Regarding the underlying cardiac diseases, there were no significant differences in the correspondence rate between short RT areas and the ablation targets across the groups [Ischemia: 8 (89%), dilated cardiomyopathy: 3 (50%), arrhythmogenic right ventricular cardiomyopathy: 0 (0%), congenital heart diseases: 3 (100%), sarcoidosis: 2 (100%), amyloidosis: 1 (50%), and hypertrophic cardiomyopathy: 1 (100%), *p* = .285].

In cases where VTs were mapped during a hemodynamically stabilized state, DZs were compared with regions of short RT based on their positional differences within the VT circuit. Of the 16 DZs corresponding to the ablation targets, four DZs (25%) exhibited complete concordance with the mid‐portion of the isthmuses in VT circuits, while the remaining 12 DZs (75%) were located near the entrance site. In contrast, four regions of short RT (16%) showed over 50% concordance with the mid‐portion of the VT isthmuses, whereas the remaining 21 regions of short RT (84%) were situated near the exit site. The median distance between the center of DZs and the center of the short RT area was 16 mm (interquartile range: 6.5–27.5 mm).

**FIGURE 4:**
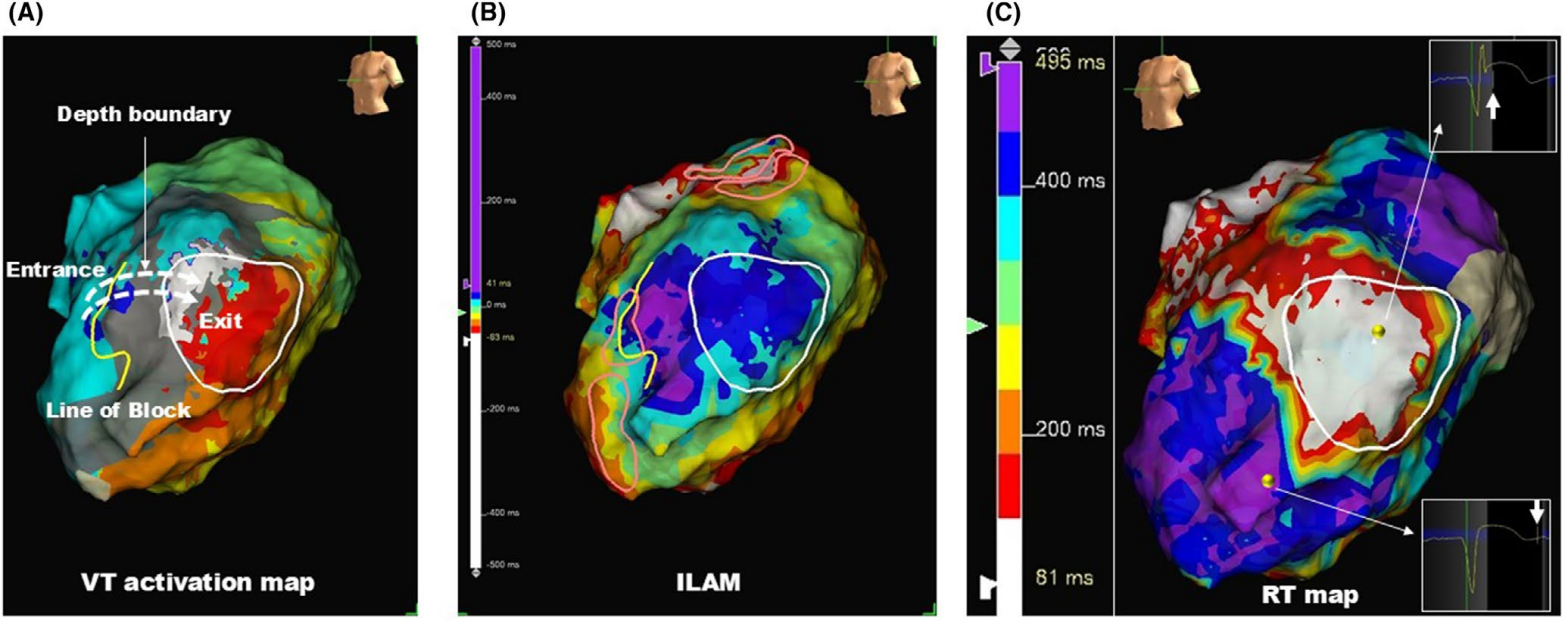
A representative case of the spatial relationship between the VT isthmus, the DZ, and the short RT area. (A) Activation map of scar‐related VT. (B) The DZs identified by ILAM are represented as pink circles. (C) RT map; regions with short RT are indicated in white. Yellow lines denote the line of block, and the white circles represent the areas where diastolic potentials were recorded during the VT. DZ, deceleration zone; ILAM, isochronal late activation map; RT, repolarization times; VT, ventricular tachycardia.

Figure 4 illustrates a representative case of scar‐related VT in a patient with ischemic cardiomyopathy, demonstrating the presence of DZs in the entrance region and a short RT region in the exit region. A line of block is shown in the anterior septum, with scar areas identified extending toward the lateral wall from this line (Figure 4A). The area marked by the white circle represents the region where diastolic potentials were recorded during the VT. Based on this color map, the VT circuit was inferred to involve VT activation propagating from the septum, entering the depth boundary from the line of block, and returning to the endocardium in the region characterized by white or red coloration. As shown in Figure 4B, the DZs indicated by the pink circles were located at the entrance site. Furthermore, the regions with short RT were located at the exit site in the lateral wall (Figure 4C).

### 3.5 Impacts of ablating areas with short RT on VT recurrence

**FIGURE 5:**
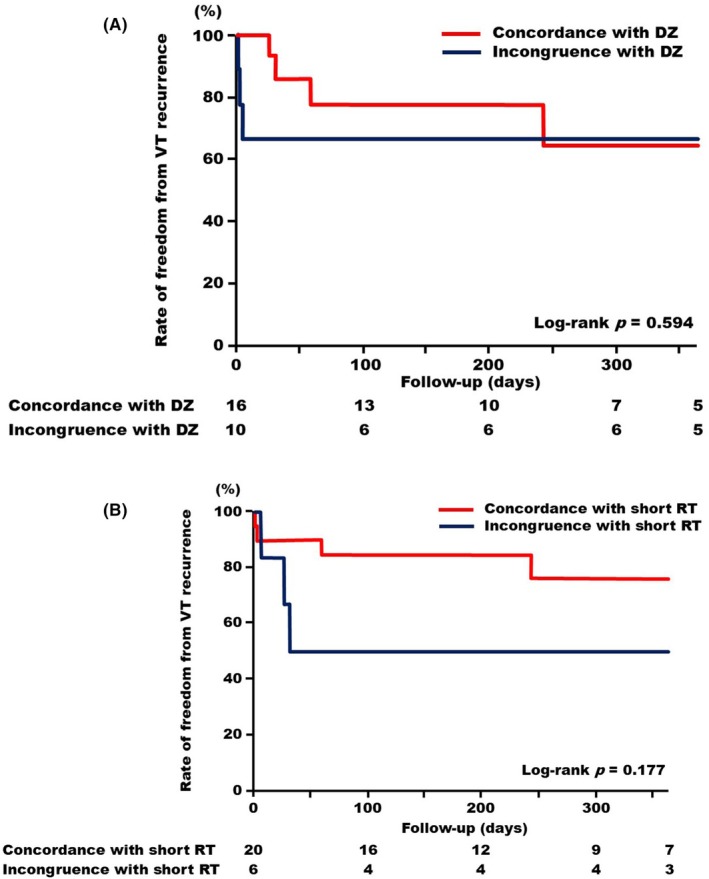
Kaplan–Meier curves depicting freedom from VT recurrences. (A) A red line indicates subjects in whom the regions targeted for catheter ablation were concordant with DZs; a blue line indicates those incongruent with DZs. (B) A red line indicates subjects in whom the regions targeted for catheter ablation were concordant with the short RT area; a blue line indicates those incongruent with the short RT area. DZ, deceleration zone; RT, repolarization times; VT, ventricular tachycardia.

The recurrence of VTs was evaluated during the 1‐year period following the procedures. The cases in which the regions targeted for catheter ablation were concordant with the DZs exhibited comparable recurrence rates to those in which the regions targeted for catheter ablation were incongruent with the DZs (Log‐rank *p* = .594, Figure 5A). Notably, although this did not reach statistical significance, the cases in which the regions targeted for catheter ablation were concordant with the short RT areas tended to have a lower recurrence rate compared to those in which the regions targeted for catheter ablation were incongruent with the short RT areas (Log‐rank *p* = .177, Figure 5B).

## 4 DISCUSSION

This study represents the first clinical report detailing the characteristics of our recently proposed technique, RT mapping, and highlighting the differences in its utility for visualizing VT circuits compared to conventional substrate mapping. The regions of short RT exhibited comparable areas to those of DZs identified by ILAM, and these areas were smaller than those of bipolar and unipolar low‐voltage areas. Furthermore, the short RT areas demonstrated strong concordance with ablation targets, which were comparable to those of DZs. In the short RT areas, the unipolar morphologies recorded using high‐pass filter settings at 0.05 Hz exhibited an up‐sloping ST segment without a negative T wave. In contrast, the long RT showed a flat or down‐sloping ST segment with a negative T wave. The areas of short RT were primarily located at the exit site, whereas the DZs were predominantly found at the entrance site, with a distance of 16 mm between the two. Cases with ablation targets corresponding to short RT areas tended to exhibit a lower incidence of VT recurrences compared to those without.

### 4.1 Limitations of conventional mapping

One of the currently accepted techniques for arrhythmogenic substrate mapping is voltage mapping; however, its limitation due to the widespread presence of bystander areas has been well recognized, as demonstrated in our findings.^10^, ^11^ This may reduce ablation efficacy. Other approaches, including delayed potentials, decremental evoked potentials, and local abnormal ventricular activities, have also been utilized to assess conduction delay. However, technical challenges related to pacing rates and coupling intervals remain.^13^ To address these issues, Tung and colleagues proposed ILAM to emphasize regions of slow conduction velocity.^1^ DZs are characterized by isochronal crowding of propagation within a scar. Komatsu and colleagues further demonstrated that the propagation of rotational activation patterns, indicative of slow conduction and considered synonymous with DZs, localizes at the entrance of the VT circuit.^14^ These methods could provide evidence for the involvement of conduction delay in the VT isthmus. However, several experimental studies have shown that the VT isthmus consists of both zones of slow conduction and regions with heterogeneous RT.^15^, ^16^ Nevertheless, the heterogeneity of the repolarization phase has not been extensively discussed in clinical practice.

### 4.2 How RT mapping addresses these issues

Our newly developed functional substrate mapping technique, namely RT mapping, based on unipolar morphologies in a high‐pass filter setting with a cutoff of 0.05 Hz, is expected to offer new insights into the reentrant circuit. Our results demonstrated that the areas of short RT were statistically comparable in size to those of the DZs identified by ILAM, with a similar correspondence rate to ablation targets for both. This finding is significant, as most VT isthmuses are located in low‐voltage areas, and the homogenization of these areas is effective for treatment.^17^ However, the presence of numerous bystander regions may extend the procedural duration. In contrast, RT mapping can help refine the areas of interest more effectively.

The positions of DZs and short RT areas within the reentry circuit are also of electrophysiological interest. Our results demonstrated that the area of short RT tended to be located adjacent to the exit site of the VT circuit, in contrast to the DZ, which was situated at the entrance site. A study on repolarization heterogeneity, referred to as the reentry vulnerability index, reported that a low reentry vulnerability index, considered synonymous with a short RT, indicates the exit site in patients undergoing VT ablation.^18^ Our findings are consistent with those of previous studies.

A key advantage of the RT map is its ability to facilitate the comparison of conduction delays identified by ILAM and RT heterogeneity through re‐mapping within a single acquired map, thereby allowing the assessment of both entrance and exit sites within the same case. The median distance between the DZ and the short RT area, referring to the entrance and exit of the VT circuit, is also important for understanding the reentrant circuit. Tung and colleagues demonstrated that the length of the boundary in the VT isthmus ranged from 10 to 20 mm, which corresponds to our finding of a median distance of 16 mm.^19^

### 4.3 Clinical significance

It is an important finding that over 70% of ablation targets corresponded to the short RT area in cases where the DZ was not identified. Given that many studies have demonstrated the efficacy of ablation targeting the DZs, we also propose that the identification of DZs should be prioritized. However, in cases without any identified DZs or where VTs remain inducible even after ablating the DZs, RT mapping should be considered.

The RT map is characterized by the use of unipolar potentials, which reflect both near‐ and far‐field signals. In contrast, bipolar potentials, employed in conventional functional substrate mapping to identify conduction delays, reflect only near‐field signals.^20^ This differentiation may be pivotal for identifying the intramural isthmus. For instance, in the third case (Figure 4), cases exhibiting a depth boundary that complicates the identification of DZs on endocardial surface mapping may benefit from assessing RT heterogeneity using the RI map to accurately identify ablation targets.

### 4.4 Technical considerations

Child et al. investigated an index derived from calculations, referred to as the reentry vulnerability index.^18^ This method identifies the VT isthmus by assessing the differences in activation time and RT between adjacent tissues using extra‐stimulation. Over 70% of VT origins corresponded to low reentry vulnerability index sites, indicating that the time derived by subtracting RT from activation time is short.^21^ This method is beneficial for evaluating tissues that can facilitate reentry through functional block; however, a critical drawback is its reliance on the nominal filter settings of the three‐dimensional mapping system when assessing unipolar electrograms. It has been noted that the high‐pass filter set at 2 Hz in the nominal settings of the three‐dimensional mapping systems significantly affects the ST segment waveform, which is crucial for determining the endpoint of the RT.^22^, ^23^

To overcome this limitation and automatically determine the endpoint of the RT, we modified the high‐pass filter of the EnSite system to 0.05 Hz. Furthermore, it is well established that the J‐ST level fluctuates due to catheter contact pressure; therefore, a grid‐like multielectrode catheter was used to minimize J‐ST level variations due to contact pressure.^24^ These two points are essential considerations for techniques that evaluate unipolar electrograms.

The primary issue with the RT map lies in the fact that while the endpoint determination is fully automated, the starting point of the “window”—specifically, the end of the QRS wave—must be manually set by the user. In certain cases, local delayed potentials, typically recognized as delayed potentials in bipolar signals, may persist beyond the QRS offset on the body surface. In such instances, if the starting point is defined at the QRS offset, the maximal *dV*/*dt* corresponding to the fractionated potentials reflected on unipolar leads may be misidentified, leading to an erroneous estimation of the actual RT duration. Therefore, the differences in blind periods can be observed between cases of hypertrophic cardiomyopathy, as shown in Figure 2 (a representative case of a short blind period), and arrhythmogenic ventricular cardiomyopathy, as illustrated in Figure 3 (a representative case of a long blind period).

### 4.5 Future research

In our data, although there was no statistically significant difference in the alignment of the short RT regions with the ablation target across underlying cardiac diseases, it is important to note that in arrhythmogenic right ventricular cardiomyopathy, there was no case where the short RT region aligned with the ablation target. Similar findings have been reported in previous studies, in which RT mapping failed to identify the VT isthmus in cases of arrhythmogenic right ventricular cardiomyopathy.^25^ Nagase et al. previously reported reduced repolarization heterogeneity when evaluating unipolar potentials in arrhythmogenic right ventricular cardiomyopathy compared to Brugada syndrome.^26^ The etiology‐specific dispersion of RT warrants further investigation.

Although it did not reach statistical significance, ablation of the short RT area demonstrated a trend toward greater effectiveness in suppressing VT recurrence compared to cases where ablation of the short RT area was not performed (Figure 5). These findings may suggest the clinical benefits of ablation targeting the exit, even in cases where conduction delay is not observed in surface mapping due to the presence of intramural conduction delay. Further clinical studies are warranted to investigate ablation targeting of short RT areas.

### 4.6 Limitations

First, as described in the Section 4.4, recordings obtained with excessive contact force can induce injury currents, leading to J‐ST elevation in the unipolar potentials. Therefore, unipolar potential recordings from the distal electrode of an ablation catheter are not suitable for evaluating RT. Second, local potentials in regions with a short RT exhibited an up‐sloping ST segment without a negative T‐wave, resembling monophasic action potentials. The determination of the terminal point of RT remains controversial in such waveforms due to the measurement of an excessively short RT; however, previous studies have demonstrated the accuracy of the Wyatt method regardless of T‐wave morphology.^6^, ^27^ Third, the changes in RT before and after ablation were noteworthy; therefore, further investigations regarding post‐ablation mappings are warranted. Fourthly, although not evaluated in this study, assessing the effective refractory periods alongside the RT map would likely enhance the credibility of the repolarization heterogeneity displayed on the RT map. Finally, as this study was conducted as a retrospective cohort registry, the clinical impacts of RT mapping should be evaluated in a multicenter prospective study.

## 5 CONCLUSIONS

Our proposed functional substrate electroanatomical mapping technique, the RT mapping, visualized the ablation targets. The short RT area, where unipolar morphology exhibited an up‐sloping ST segment without a negative T wave, was indicative of the exit site in the VT circuit. Considering both repolarization heterogeneity and conduction delay is expected to provide a comprehensive understanding of the critical isthmuses involved in scar‐related VT.

## FUNDING INFORMATION

This work was partly supported by a grant from the Yomiuri Television Charity Fund.

## CONFLICT OF INTEREST STATEMENT

Authors declare no conflict of interests for this article.

## ETHICS APPROVAL STATEMENT

This study adhered to institutional ethics and integrity guidelines. The study was approved by the institutional review board (Number: R2017071).

## PATIENT CONSENT STATEMENT

Informed consent was waived given the retrospective nature without any intended interventions and opt‐out method.

## Data Availability

The data supporting the findings of this study are available from the corresponding author upon reasonable request.
